# Is there an Alternative to TIPS? Ultrasound-guided Direct Intrahepatic Portosystemic Shunt Placement in Budd-Chiari Syndrome

**DOI:** 10.4103/1319-3767.70633

**Published:** 2010-10

**Authors:** Bora Peynircioglu, Ali Ibrahim Shorbagi, Omur Balli, Barbaros Cil, Ferhun Balkanci, Yusuf Bayraktar

**Affiliations:** Department of Radiology, Hacettepe University, School of Medicine, Ankara, Turkey; 1 Department of Gastroenterology, Hacettepe University, School of Medicine, Ankara, Turkey

**Keywords:** Budd-Chiari syndrome, portal hypertension, transjugular intrahepatic portosystemic shunt, direct intrahepatic portosystemic shunt

## Abstract

Budd-Chiari syndrome is a spectrum of manifestations which develops as a result of hepatic venous outflow obstruction. Transjugular intrahepatic portosystemic shunt (TIPS) is a minimally invasive vascular and interventional radiological procedure indicated in the management of refractory ascites in such patients. Conventional TIPS requires the presence of a patent hepatic vein and reasonable accessibility to the portal vein, and in patients with totally occluded hepatic veins, this procedure is technically challenging. Direct intrahepatic portosystemic shunt (DIPS) or so called “percutaneous TIPS” involves ultrasound-guided percutaneous simultaneous puncture of the portal vein and inferior vena cava followed by introduction of a guidewire through the portal vein into the inferior vena cava, as a deviation from conventional TIPS. Described here is our experience with DIPS. Three patients with BCS who had refractory ascites but were unsuitable for conventional TIPS due to occlusion of the hepatic veins were chosen to undergo the DIPS procedure. Our technical success was 100%. The shunts placed in two patients remain patent to date, while the shunt in a third patient with underlying antiphospholipid syndrome was occluded a month after the procedure. The percutaneous TIPS procedure seems to be technically feasible and effective in the management of refractory ascites as a result of BCS, particularly in the setting of occluded hepatic veins.

Budd-Chiari syndrome (BCS) is a spectrum of clinical presentations which develop as a result of hepatic venous outflow obstruction. Ensuing acute hepatic parenchymal congestion and inflammation eventually lead to liver dysfunction and cirrhosis. A hypercoagulability state remains the most common cause of the disorder,[1] therefore warranting investigation for risk factors that potentiate venous thrombosis, or thrombophilia.[2] Untreated BCS has a mortality rate of 80%.[3] Available treatment options for BCS include TIPS, membranotomy, radical resection of the thrombus, surgical shunts, and liver transplantation.[4] TIPS, as a relatively less invasive interventional radiological modality, has over the years proven itself as a feasible alternative to surgery. The TIPS procedure has two critical requirements: the presence of a patent hepatic vein and reasonable accessibility to the portal vein. Nevertheless, several alternative TIPS techniques have been developed in case of occluded hepatic veins, including attempts to connect the portal vein to a hepatic venous stump or even hepatic vein recanalization. The gun-sight technique was first introduced in 1996 as a fluoroscopy-guided transcaval technique which was then modified to a direct intrahepatic porto-systemic shunt (DIPS) in 2006, as an alternative to TIPS.[5–7] Presented here is our experience with this novel technique in three patients with BCS who had completely occluded hepatic veins.

## CASE SUMMARIES

Of the patients with BCS who were referred to the Vascular and Interventional Radiology Unit for refractory ascites between December 2005 and February 2009, three were selected for percutaneous intrahepatic porto-systemic shunting [Table 1].

**Table 1:** Summary of the three cases with BCS who underwent DIPS at Hacettepe University

Age/sex	Underlying disorder	Procedure date	Post-procedural complication	Follow-up	Patency period
42/M	Idiopathic	12/2005	Early shunt obstruction (Day 3)	Shunt revision with placement of stent-graft as well as a Wallstent to the compressed segment of the IVC	40 months*
20/F	Anti-phospholipid syndrome	08/2008	None	Developed HIT, preventing initiation of coumadine. Shunt occlusion with 1 month.	1 month
50/M	Polycythemia vera rubra	02/2009	None	Progressive decrease in ascites; improvement of renal function (hemodialysis was discontinued)	3 months*

HIT: heparin-induced thrombocytopenia.

* Shunts still patent to date of article submission. Periods calculated up to May 2009

## DIPS TECHNIQUE

**Figure 1:**
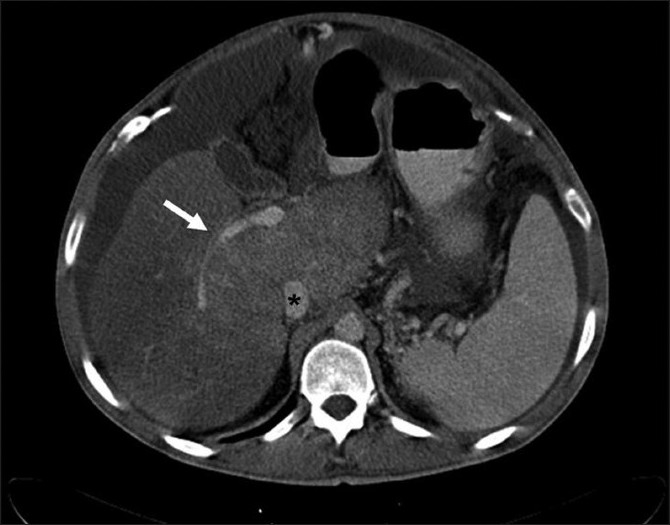
Portal venous phase image of the abdominal CT series of patient number 3 showing the relation between the right portal vein (white arrow) and inferior caval vein (*). Also note that the course of the percutaneous needle was more-or-less similar to the course of the arrow in the caudacranial direction. 187×187mm (150 × 150 DPI)

**Figure 2:**
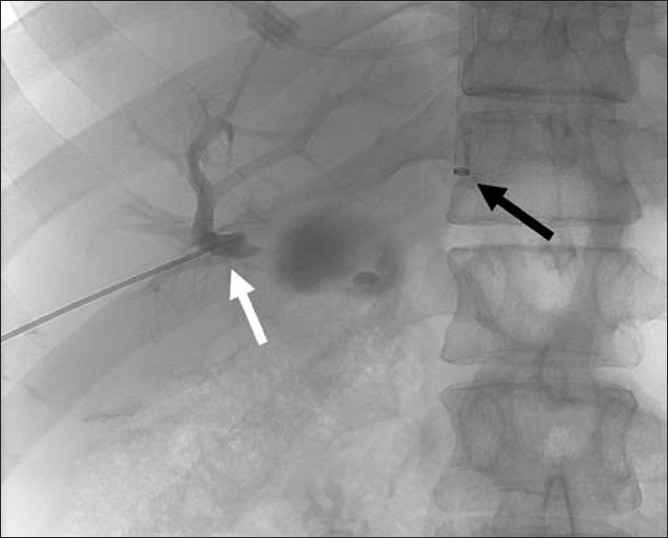
Single shot image during the percutaneous TIPS procedure demonstrating the peripheral intrahepatic puncture of right portal vein (white arrow) with contrast injection. Black arrow is pointing at the introducer sheath at the inferior vena cava placed through the left internal jugular vein. 125 × 144 mm (150 × 150 DPI)

**Figure 3:**
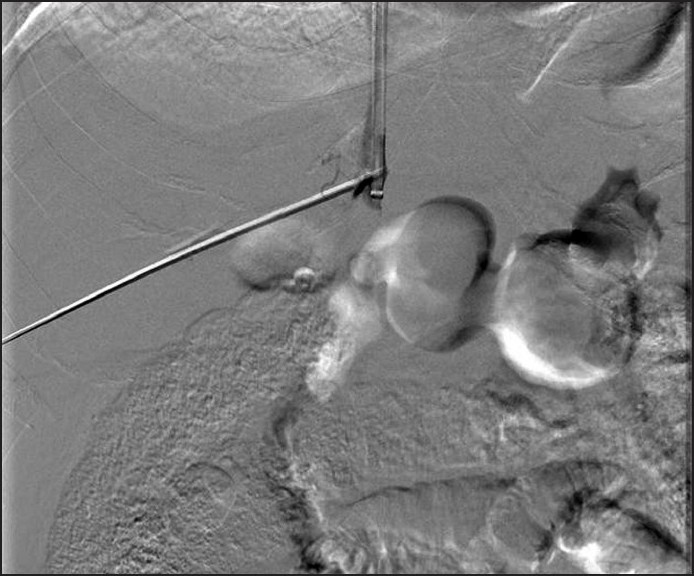
Digital substraction angiography image during contrast injection via the percutaneously placed needle coursing through the right portal vein confirming successful introduction into the inferior vena cava. 185 × 185 mm (140 × 140 DPI)

**Figure 4:**
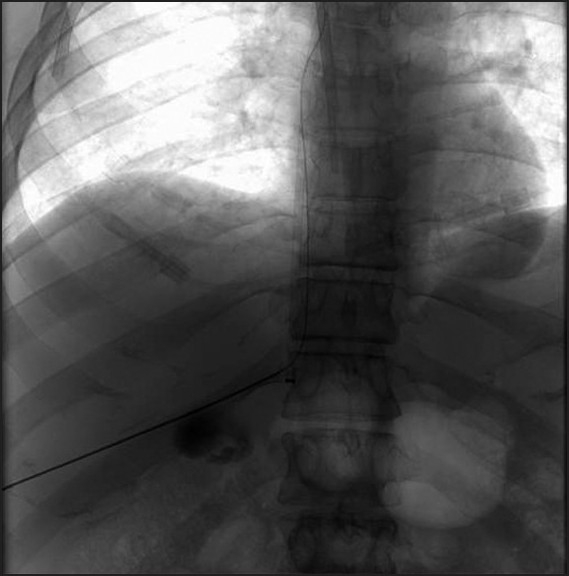
Single shot image showing the wire advanced through the percutaneous needle up to the right atrium. Also note the dialysis catheter within the right atrium which was placed through the right internal jugular vein. 188 × 188 mm (138 × 138 DPI)

**Figure 5:**
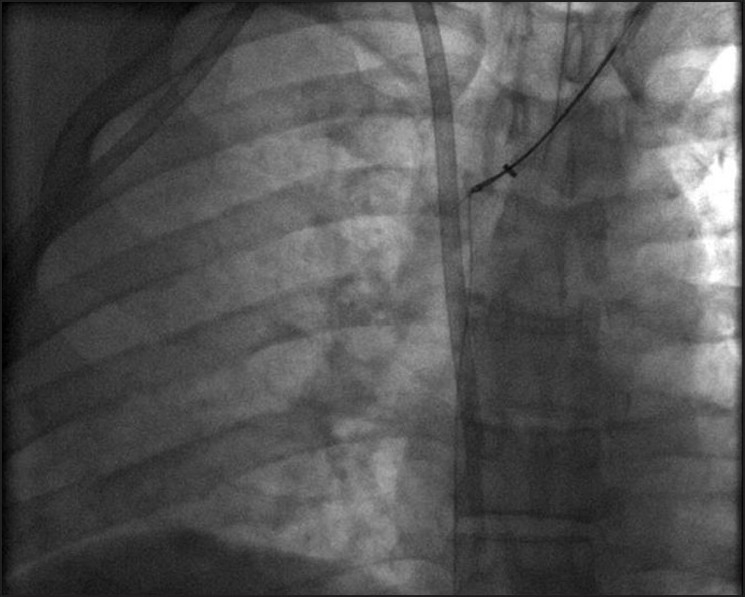
The wire advanced through the needle is now snared by a goose neck snare placed using the left internal jugular vein approach. 190 × 190 mm (137 × 137 DPI)

**Figure 6:**
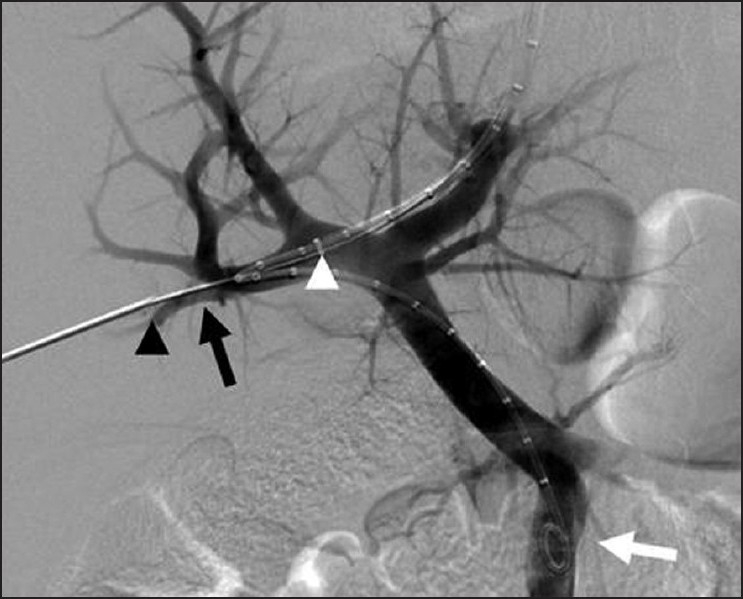
The snared wire (black arrow) is now used as a “through and through” access from the percutaneous transhepatic needle to the left internal jugular vein. The needle tip (black arrow head) is retracted just proximal to the right portal vein entry. The tip of the introducer sheath (white arrow) placed via the left internal jugular vein is now advanced over the “through and through” wire into the right portal vein. A graduated pigtail catheter (white arrow) is also advanced through the introducer sheath next to the “through and through” wire and directed to the superior mesenteric vein via the main portal vein using standard angiographic techniques. 113 × 125 mm (150 × 150 DPI)

**Figure 7:**
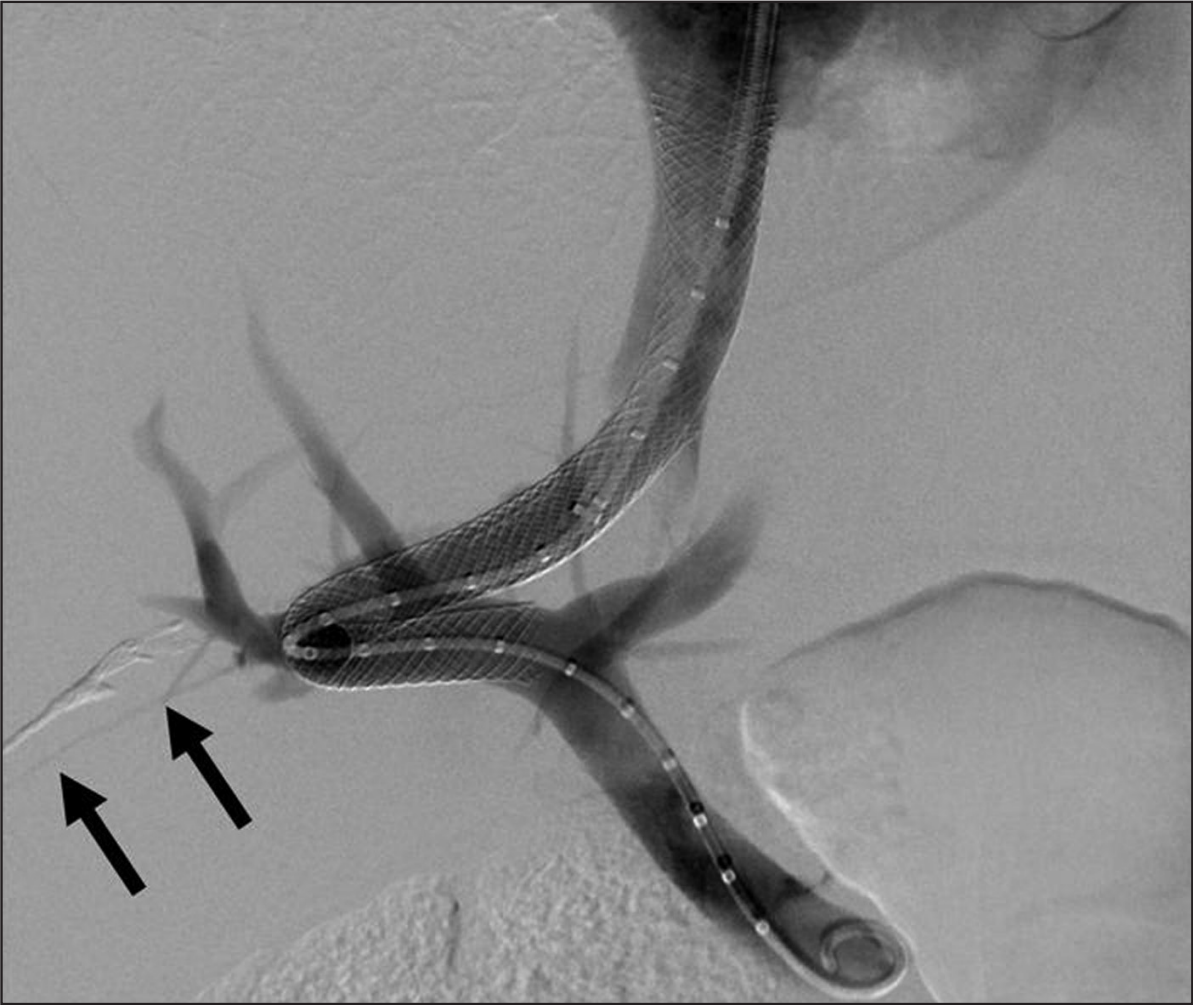
Contrast injection after TIPS placement showing widely patent porto-systemic shunt with good flow to the right atrium and diminutive filling of the intra-hepatic portal vein branches. Also note the glue cast injected to the needle tract in order to prevent bleeding to the abdominal cavity filled with ascites. 189 × 189 mm (150 × 150 DPI)

Prior to the procedure patients are evaluated by both Doppler ultrasound and abdominal computed tomography for patency of the hepatic veins and to establish the anatomy of the venous system of the liver. For this percutaneous TIPS technique, jugular access is again utilized; however, as a deviation from conventional TIPS, the path of the shunt that is due to be placed between the portal vein and the inferior vena cava is determined by introducing an ultrasound-guided needle percutaneously via the abdominal wall somewhere along the anterior axillary line. Although general anesthesia was preferred in all our patients, deep intravenous sedation with local anesthesia can also be used as standard TIPS procedures. Sterilization of the neck and anterior/lateral abdomen using an iodine solution is performed in a standard manner. After a vascular access sheath is placed into the internal jugular vein, pressure measurements of the right atrium are performed to rule out the presence of right heart failure. Percutaneous transhepatic puncture is performed using an 18 or 21 gauge Chiba needle. Total paracentesis before the procedure has been proven to decrease the risk of complications, and this was performed in two of our patients. Dynamic abdominal CT obtained prior to the procedure is really helpful in determining the portal and inferior caval vein relation and thus percutaneous puncture site with appropriate angle to create an optimal shunt [Figure 1]. The Chiba needles are guided under ultrasound towards the anterior branch of the right portal vein, after which entry into the portal vein is confirmed by injection of contrast material [Figure 2]. Without changing the angle of entry, the needle is introduced further toward the inferior vena cava, again under the guidance of ultrasound. Entry into the IVC is confirmed by contrast material injection [Figure 3]. A 0.018 or 0.035 inch guidewire is then introduced through the Chiba needle until the right atrium is reached [Figure 4]. “Through-and-through” access is then achieved by pulling the tip of the guidewire into the right atrium through the vascular sheath and out using a snare [Figure 5]. The guidewire now passes from the internal jugular vein to the inferior vena cava, through the liver parenchyma, into the portal vein and then out through the anterior abdominal wall. From here on the same maneuvers that are used in conventional TIPS to place the stent and/or stent/graft are utilized, with the only difference being access through the IVC instead of the hepatic veins [Figure 6]. Bare metallic stents were used initially in all of our patients. Direct portography and pressure measurements are made before and after placement of the shunt, and post-dilatation is performed until optimal pressure levels and portography findings are achieved [Figure 7]. To decrease the risk of bleeding, the transhepatic tract that extends to the abdominal wall from the portal vein puncture site is embolized using a liquid embolizing agent as the needle is being removed [Figure 7]. Afterward, patients are started on intravenous heparin, and depending on the underlying thrombophilic disorder, treatment with coumadine is initiated within 24 h after any signs of bleeding have been ruled out. Follow-up Doppler US is performed on days 1 and 7, then monthly for 3 months, after which 3-monthly evaluations are done.

## RESULTS

Technical success with our patients was 100%. In all patients, who were particularly selected for the DIPS procedure for having completely occluded hepatic veins, placement of a shunt between the inferior vena cava and the right portal vein was successful. All shunts created were 10 mm in diameter according to the body weights of the patients. All patients recovered well after the procedure, and were ready for discharge from the hospital within 24 hrs with a plan of long-term anticoagulation therapy.

No minor or major complications occurred during or after the procedures except for early shunt occlusion in Case 1 with idiopathic BCS. This was attributed to biliary leakage and the presence of a severely compressed intrahepatic section of the inferior vena cava. A stent-graft had to be placed within the shunt 3 days after the DIPS procedure, and additonal stenting of the inferior vena cava was performed to relieve caval drainage. To date, the patient’s revised shunt remains patent with his ascites under control.

Case 2 who had antiphospholipid syndrome developed heparin-induced thrombocytopenia requiring discontinuation of infusion within the first 24 hrs after the procedure. The thrombocytopenia was deemed a contraindication for coumadinization. She was instead started on the heparin analogue fondaparinux. Unfortunately, shunt occlusion was detected at 1 month follow up by Doppler ultrasound with deterioration of her clinical condition (marked ascites and lower extremity edema). Further intervention was not considered for this patient. She eventually died following a thrombotic cerebrovascular incident.

Case 3 with polycythemia rubra vera, who was also oliguric, had significantly increased urinary output 2 weeks after the procedure. During the following 3 months, ascites progressively regressed, and improved renal functions alleviated the need for hemodialysis. Doppler ultrasound evaluation revealed a patent shunt with a moderate level of ascites at follow-up.

Cases 1 and 3 remain on anticoagulation therapy with coumadine, and both had patent shunts by the date of submission. All three cases have been summarized in Table 1.

## DISCUSSION

The first TIPS procedure on a BCS patient was performed by Peltzer in 1993.[8] In BCS, TIPS has proven itself to be an effective method at decompressing the congested liver, decrease portal venous pressure, improving liver functions, and controlling ascites, especially since the procedure is technically not affected by an enlarged caudate lobe.[5] The most critical and difficult aspect of the TIPS procedure is access to the portal vein. With conventional TIPS, blind efforts in accessing the portal vein through the hepatic vein may lead to complications as well as prolonging fluoroscopy time. Percutaneous TIPS, otherwise known as direct intrahepatic porto-systemic shunt (DIPS), was first introduced in 1996 as a gun-sight technique bearing in mind the above-mentioned difficulties.[5,6] The simultaneous portal vein and inferior vena cava entry method employed with DIPS allows for completion of the TIPS procedure without requiring a second vascular access, while also proving to be very effective. The percutaneous TIPS procedure seems to be technically feasible and effective in the management of refractory ascites as a result of BCS, particularly in patients who are not suitable for standard TIPS procedure. Early coumadinization is essential for long-term success in this patient group, especially when several underlying thrombotic disorders are taken into consideration.
